# Supplementary material for: Ang II-Induced Hypertension Exacerbates the Pathogenesis of Tuberculosis
Source: Cells. 2021 Sep 19;10(9):2478. doi: 10.3390/cells10092478 (PMC8465031; doi:10.3390/cells10092478)
Supplement: Supplementary file 1 [file cells-10-02478-s001.zip › cells-1360060-supplementary.pdf]

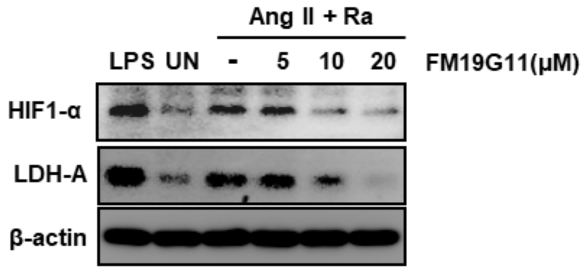

Supplementary Figure S 1. Ang II-induced LDH-A production is dependent on HIF1-α production during *Mtb* infection. BMDM cells were pretreated with FM19G11 (HIF1-α modulator, 5–20 μM) for 1 hour and were incubated with Ang II (1 μM) during *Mtb* infection (MOI = 1). Western blotting analysis was performed to examine the expression levels of HIF1-α, LDH-A protein at 24 hours after infection. LPS (500 ng/mL, 24 hours) was used as a positive control.
